# Maximum Power Efficiency

**DOI:** 10.3390/e27070714

**Published:** 2025-07-01

**Authors:** Boye Ahlborn, Frank Curzon

**Affiliations:** Department of Physics and Astronomy, University of British Columbia, Vancouver, BC V6T 1Z3, Canada; curzon@phas.ubc.ca

New research often starts with vague, dream-like ideas, conversed on over coffee in the free flow of animated discussions about physics, the growing up of one’s children, politics, and the success of the local ice hockey team.

Neither of us—Frank Curzon and myself—had any theoretical aspirations. Frank lectured on an E&M course and his students worked on Z-pinch experiments, and my students worked on pressure- or energy-driven plasma flow, like shock waves, plasma jets, and chemical detonations; I was just preparing lecture notes on thermodynamics.

One day, I casually mentioned that the Carnot efficiency is just an energy budget, one that has nothing to do with the operation of an engine that delivers mechanical power. Frank picked this casual comment up. He liked puzzles and played with physics relations for amusement, and he found solutions to questions that had not been asked before.

The authors in 1975.


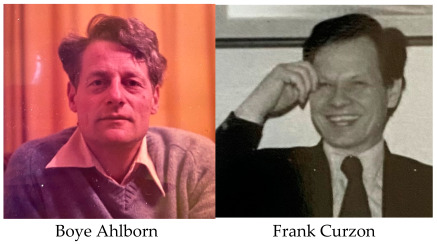


After a few days, Frank came back with the observation that my hunch actually led to an optimum for the energy conversion when energy flows into, and out of, a heat engine at a certain rate [1]; namely, the energy transfer velocity v_rms_, or the root mean square velocity of a gas in thermal equilibrium.

(1) $$v_{rms}=\sqrt{3kT_{D}/m_{b}},$$

The efficiency at this maximum is

(2) $$\eta_{max}=1-\sqrt{T_{cold}/T_{hot}},$$

and the square root contains the ratio of energy transfer velocities:

(3) $$\sqrt{T_{cold}/T_{hot}}=\frac{v_{rms,cold}}{v_{rms,hot}}$$

Instead of the rms velocities, the average velocity, or the maximum of the velocity distribution could be used, since these velocities differ from the rms velocity only by a numerical constant, which drops out in the ratio of T_cold_/T_hot_.

Equation (2) is clearly different from the energy bookkeeping of the Carnot efficiency, and we chose the name “maximum power efficiency” in a short research note—which was rejected by two different journals. To us, however, this result looked too straightforward to be wrong, and it came as a relief when the American Journal of Physics let it go into print, although the referee noted that “while he could see nothing wrong with the derivation he had doubts about the relevance of this result”.

The root mean square velocity, Equation (1), connects thermodynamic and mechanic parameters that otherwise live independent of each other in different branches of physics: temperature has neither time nor distance, and mechanical motion has no association with temperature, but both deal with energy. The gap between them is closed by fundamental physics constants and by the conservation of energy. In the case of the efficiency calculations, the Boltzmann constant, k = 1.38 × 10^−23^ [J/°K], is the missing link, which also appears in the gas law

p = NkT,(4)

Connections between temperature, T, and energy also appear in radiation laws, like Stephan’s law, which gives radiation intensity, W, as function of the temperature of a “black body”.

W = σT^4^, [watt/m^2^K], (5)

Here, the connection comes in through the constant σ = 5.67×10^−8^ [K^4^], and in Wien’s displacement law for the maximum wavelength, λ_max_, of a blackbody spectrum

λ_max_T = b = 2.98 × 10^−3^ [m·K] (6)

The parameters b, k, and σ are derived from the statistical mechanics of the random velocity distributions of particles in local thermal equilibrium. Actually, at low densities, a plasma can have “hot” electrons, where T_el_ > T_ion_ and each group is in local thermal equilibrium.

Energy is a unique parameter that appears in all branches of physics, including fluid dynamics (plasma physics). This can be seen in the conservation of energy for a one-dimensional flow, driven by the input of some energy Q$\left[\frac{J}{kg}\right]=\left[\frac{m^{2}}{s^{2}}\right]$ which is equal to the sum of all forms of energy per unit mass (not including electrodynamic energies): the enthalpy increase ∆h = ∆p + ∆u (pressure p + internal energy u), the generation of kinetic energy per unit mass ½v^2^, and the energy lost or gained by field in terms of W [watt/m^2^] of radiation or sound:

(7) $$Q\left[\frac{J}{kg}=\frac{m^{2}}{s^{2}}\right]=∆h\left[\frac{J}{kg}\right]\left(T\right)+\frac{1}{2}v^{2}\left[\frac{m^{2}}{s^{2}}\right]+\frac{W\left(T\right)\left[\frac{watt}{m^{2}}\right]}{\rho_{1}v_{1}\left[\frac{kg}{m^{2}}\right]}$$

Some examples of such flow are rockets, detonations, plasma jets, and even astrophysical radiation fronts. Another overriding principle is the conservation of mass flow J, of density ρ [kgm^3^], and velocity v [m/s]: What flows in ρ_a_v_a_ must come out ρ_b_v_b_:

J = ρ_a_v_a_ =ρ_b_v_b_ = const [kg/sm^2^] (8)

Equation (7) is used to refer the field energy W to the mass flow J, thus generating the unit J/kg. Between Q and any two of these energy forms, a conversion efficiency could be defined.

A “hunch” is like a spark that flashes up when steel hits the grinding wheel, and lights a fire in nearby fuel. Finite time thermodynamics was certainly not in sight at the time of our morning coffee efficiency-speculations. Obviously, the impact of a communication does not depend on the size of the research program, but rather on taking an unconventional look at existing questions.
